# The Prevalence and Effect of Cosmetic Procedures on Patients with Rheumatic Diseases: A Cross-Sectional Survey

**DOI:** 10.3390/healthcare14030378

**Published:** 2026-02-02

**Authors:** Ibrahim Almaghlouth, Haya M. Almalag, Reema Bader AlEnezy, Sarah AlEnezy, Rahaf Althnayan, Munira Abdulrahman Alhadlg, Hajer Alzuhair, Rafif Alsaigh, Asma Bedaiwi, Lena M. Hassen, Sulaiman Alzomia, Boshra Alanazi, Saud Alahmari, Abdulaziz M. Abdulkareem, Kazi Nur Asfina, Hebatallah H. Ali, Najma Khalil, Mohammed A. Omair, Mohamed Bedaiwi, Lama R. Alzamil, Abdulaziz Madani, Abdurhman S. Alarfaj

**Affiliations:** 1 Rheumatology Unit, Department of Medicine, College of Medicine, King Saud University and Medical City, Riyadh 11451, Saudi Arabia; ialmaghlouth@ksu.edu.sa (I.A.); alenezyreema@gmail.com (R.B.A.); s.alenezy1@gmail.com (S.A.); rahaf.althnayan@gmail.com (R.A.); dr.hajeralzuhair@gmail.com (H.A.); lenamhassen@gmail.com (L.M.H.);; 2 Department of Clinical Pharmacy, College of Pharmacy, King Saud University, Riyadh 11451, Saudi Arabia; 3 Department of Dermatology, College of Medicine, King Saud University, Riyadh 11451, Saudi Arabia; munira.alhadlg@gmail.com (M.A.A.);; 4 Zoology Department, College of Sciences, King Saud University, Riyadh 11451, Saudi Arabia; 5 College of Medicine Research Centre, College of Medicine, King Saud University, Riyadh 11451, Saudi Arabia

**Keywords:** arthritis, rheumatoid, plastic surgery procedures, cross-sectional studies, surveys and questionnaires

## Abstract

**Objective:** Due to the increasing prevalence of rheumatological conditions worldwide, especially among women, and their known negative impact on body image, there is a growing demand for cosmetic procedures. Therefore, it is imperative to develop an evidence-based understanding of the safety of these procedures and their potential effects on the disease course to prevent undesirable exacerbations. **Methods**: An observational cross-sectional survey was conducted among adult patients diagnosed with rheumatic diseases. Data were collected using an electronic questionnaire that addressed demographics, disease characteristics, comorbidities, and perceptions of cosmetic procedures. Ethical approval was obtained from the Institutional Review Board of King Saud University and King Saud University Medical City. Appropriate descriptive and inferential statistical analyses were performed. **Results:** A total of 212 participants were included; among them, 92 participants considered or underwent cosmetic procedures, while 120 did not. A significant difference was observed between groups regarding disease-related impact on self-confidence (*p* = 0.01). Factors associated with undergoing cosmetic procedures included gender (female sex) (OR 12.02; 95% CI: 1.55–93.17; *p* = 0.017), higher educational level (OR 14.00; 95% CI: 1.32–147.42; *p* = 0.028), a monthly income of SAR 1000–5000 (OR 2.39; 95% CI: 1.03–5.53; *p* = 0.041) or SAR 5000–10,000 (OR 2.75; 95% CI: 1.19–6.33; *p* = 0.017), and employment status (OR 1.81; 95% CI: 1.03–3.18; *p* = 0.038). **Conclusions:** A substantial proportion of patients with rheumatic diseases considered or had undergone cosmetic procedures, primarily driven by appearance-related concerns and reduced self-confidence. Female sex, higher education, higher income, and employment status were significant predictors. Fear of disease flare-ups and potential side effects were the most common reasons for avoiding cosmetic procedures.

## 1. Introduction

The prevalence and impact of rheumatic disease in the Saudi region have been evaluated in several studies. The reported prevalence of rheumatic illnesses, including rheumatoid arthritis, systemic lupus erythematosus, and psoriatic arthritis, ranged from 0.18 to 1.5, with a female predominance and a female-to-male ratio of 9:1 [1,2,3,4,5].

Invasive and non-invasive cosmetic procedures are increasingly common and have a documented positive impact on body image. Patients who underwent these procedures reported greater satisfaction with their appearance and higher self-esteem afterward. Evidence from clinical research indicates that rhinoplasty, breast augmentation, and abdominoplasty are among the procedures frequently associated with higher rates of patient satisfaction and positive body-image outcomes [6,7]. Patients with connective tissue disorders (rheumatological conditions) may be at greater risk of low self-esteem and thus need support [8]. With advances in the field of cosmetic surgery, attention has been drawn to the fact that some of these procedures (particularly silicone breast implants) may be linked to the development of connective tissue disease (CTD) [9]. In addition, patients with connective tissue diseases may need to undergo cosmetic procedures due to psychological or disease-related factors [10,11]. Unfortunately, the prevalence of these procedures among patients with rheumatic disease and their relationship with disease development or worsening have not been fully investigated.

The prevalence of cosmetic procedures in KSA was most recently evaluated by Almasri et al. in 2017, with an estimated prevalence of 55.4% [4,5]. This drastic contrast could be due to a smaller sample size composed of only female university students. To confirm the most popular cosmetic procedures, a recent 2018 study by Reham Almuhaya surveyed cosmetic procedures in the Saudi region; the most frequent cosmetic intervention was Botox injection (41%), and the most frequent facial plastic procedure was rhinoplasty (59%) [12]. In 2017, Almasri et al. reported that 78.3% of their sample underwent laser procedures (e.g., hair removal, fractional resurfacing, and vascular/pigment lasers), 30.0% underwent dermal fillers, 25.3% underwent skin peels, and 14.4% underwent Botox injections [4]. In a slightly older study, Alharethy [13] reported that 30.4% of the sample underwent rhinoplasty, 26.2% underwent laser hair removal, 19% underwent Botox, 14.3% underwent liposuction, and 5.8% underwent dermal fillers. Alharethy also stated that the top five minimally invasive interventions among females included Botox injection, chemical peel, laser hair removal, microdermabrasion, and sclera therapy. At the same time, the top five surgical interventions were breast augmentation, liposuction, rhinoplasty, blepharoplasty, and abdominoplasty [12]. All these procedures were reported in normal patients, not in patients with rheumatological conditions.

Due to the relatively high prevalence of rheumatic diseases in our region and the rising demand for cosmetic procedures, especially among young females, it has become imperative that we develop a clear, evidence-based understanding of the safety of and the possible effects these procedures may elicit on the course of disease in patients in order to prevent any undesirable exacerbations.

## 2. Research Methodology

### 2.1. Study Design

This study is a mixed-methods study that includes a cross-sectional survey and qualitative analysis. The research was observational and guided by the Strengthening the Reporting of Observational Studies in Epidemiology (STROBE) checklist [14].

### 2.2. Setting

This study is part of an ongoing cohort study of patients with rheumatic diseases [15]. Eligible participants were recruited between July 2021 and January 2022. Participants were recruited from the Charitable Association for Rheumatic Diseases, the Saudi Inflammatory Disease Patients Support Group, and the Specialized Rheumatology Clinic at King Khalid University Hospital, King Saud University. The phone numbers of all registered patients (a total of 3000 patients) were accessed by database governors to send text message invitations with a link to complete our survey.

### 2.3. Participants

All eligible participants were contacted, and once they agreed to participation and consented, the link to the electronic survey was sent. To be included, participants had to be adults (>18 years of age) with at least one diagnosed rheumatic disease. Any participant who did not meet the inclusion criteria was excluded. In addition, participants who underwent cosmetic procedures to correct congenital deformities were excluded.

### 2.4. Study Variables

Study variables included the following: demographic data; information about the type and nature of the participant’s rheumatic disease; comorbidities (including both physiological and psychological); their views towards cosmetic procedures; and whether they had ever undergone any cosmetic procedures. The electronic form was piloted to ensure face validity (*n* = 10) (Supplementary Material S1).

### 2.5. Data Source and Measurements

An electronic survey was sent to participants who agreed to participate. The link was sent through messaging channels, i.e., Twitter, WhatsApp, Telegram, and traditional SMS.

### 2.6. Ethical Considerations

All surveys were anonymous, with no identifying information. Participants provided electronic consent before completing the survey. No questionnaires from other authors were used. This study was evaluated and approved by the local ethics committee, the institutional review board of King Saud University, and Medical City (IRB number E-19-3955/58). Patient identifiers will remain concealed throughout this study. Informed consent was obtained electronically prior to enrolment, in accordance with the IRB guidelines of KSU. The cohort study protocol has been registered on ClinicalTrials.gov (ClinicalTrials.gov; identifier: NCT04604990).

### 2.7. Statistical Analysis

Data were coded and entered into IBM SPSS Statistics version 28 (Armonk, NY, USA). Numerical variables were presented as mean and standard deviation or median and interquartile range, depending on the distribution. Categorical parameters were reported as numbers and percentages. Parametric and non-parametric tests were used whenever appropriate to assess correlations and differences in cosmetic procedures. Binary logistic regression was performed to predict the odds of factors influencing cosmetic procedures, including rheumatologic illness. A *p*-value of <0.05 was considered significant.

## 3. Results

### 3.1. Demographics and Cosmetic Procedure

A total of 212 patients with rheumatic conditions were included in this study. Participants had a mean (SD) age of 36 years [5]; most participants were female (92%), Saudi nationals (85%), from the central region (58%), unmarried (51%), held a college or university degree (62%), had below-average income (28%), and were unemployed (62%) (Table 1). More than half of the included participants did not consider or had not undergone cosmetic procedures (57%). To explore demographic differences between participants who considered/underwent cosmetic procedures and those who did not, data were stratified and analyzed accordingly (Table 1). There were significant differences between the two groups in sex, education, income, and employment. Patients who considered/underwent cosmetic procedures were more likely to be female (98% versus 88%, *p*-value = 0.003) and employed (46% versus 32%, *p*-value = 0.037) with a college or university education (66% versus 58%, *p*-value = 0.043) and average income (34% versus 22%, *p*-value = 0.036).

### 3.2. Diseases, Including Rheumatological Conditions, Medications, and Cosmetic Procedures

Most participants had SLE (47%), inactive disease (49%), and one or fewer flares per month (69%). The majority were receiving medication for their rheumatological illness (97%), including immunosuppressants (55%) and antimalarials (51%), but not glucocorticoids (86%). Participants did not have dermatological conditions related to the illness (52%) or other comorbidities (66%) (Table 2). The only difference in participants’ disease-related characteristics between those who considered/underwent cosmetic procedures was a lack of confidence (49% versus 32%, *p*-value = 0.011) (Table 2). The presence of mental illness and its relation to participants who considered/underwent cosmetic procedures was explored; however, no association was found.

### 3.3. Types of Cosmetic Procedures with Reasons for Considering or Undergoing

**Figure 1:**
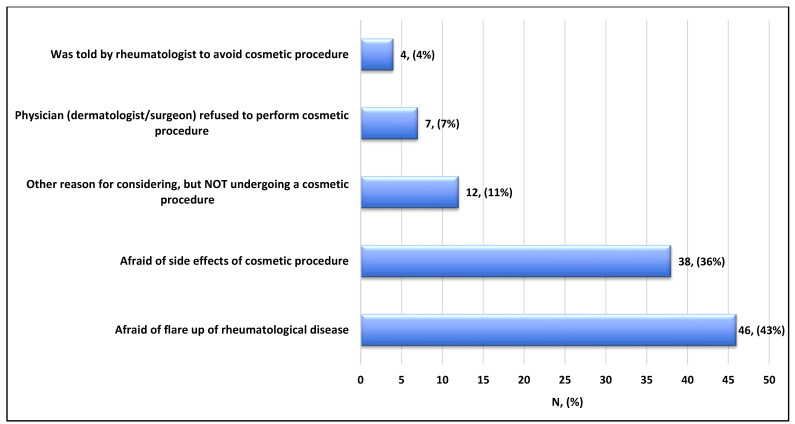
Reasons for not considering/undergoing cosmetic procedure in patients with rheumatological disorders (n = 107).

**Figure 2:**
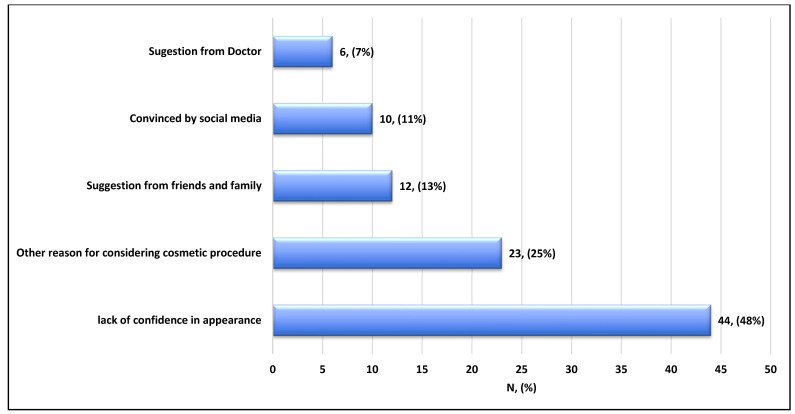
Reason for considering/undergoing cosmetic procedures in patients with rheumatological disorders (n = 92).

**Figure 3:**
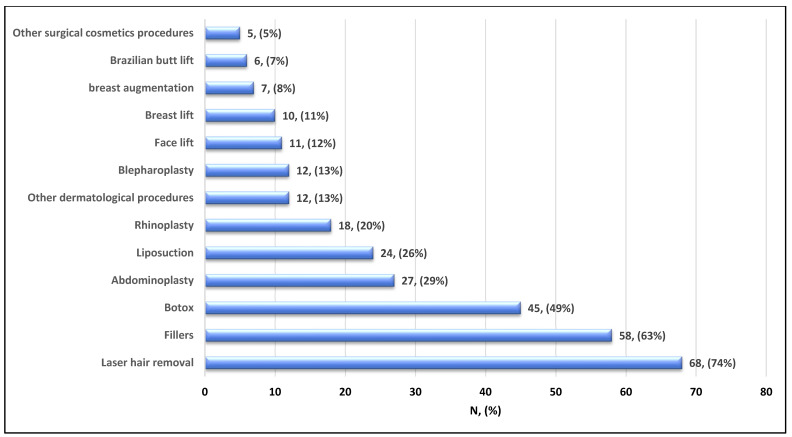
Types of cosmetic procedure considered/undergone in patients with rheumatological disorders (n = 92).

The main reason for not considering or undergoing cosmetic procedures was mainly due to fear of a rheumatologic illness flare-up (43%), followed by fear of side effects (36%) (Figure 1). On the other hand, participants who considered/underwent cosmetic procedures mostly did so due to a lack of confidence (48%), followed by other undetermined reasons (25%), and suggestions from family and friends (13%) (Figure 2). Among the most used procedures, laser hair removal topped the list (74%), followed by fillers (63%), Botox (49%), abdominoplasty (29%), liposuction (26%), and rhinoplasty (20%) (Figure 3).

### 3.4. Exploring Factors Influencing Considering or Undergoing Cosmetic Procedures

Using binary logistic regression, factors that significantly influenced the consideration or undergoing of cosmetic procedures among patients with rheumatological conditions included female sex (OR 12.019; 95% CI: 1.550–93.175; *p* = 0.017), followed by having a college or university education (OR 14.000; 95% CI: 1.329–147.429; *p* = 0.028). Average and above-average income were also associated with higher odds (OR 2.393; 95% CI: 1.035–5.535; *p* = 0.041; and OR 2.751; 95%: CI 1.195 –6.334; *p* = 0.017, respectively), as was being employed (OR 1.813; 95% CI: 1.033 –3.181; *p* = 0.038) (Table 3).

## 4. Discussion

The cutaneous manifestations of inflammatory autoimmune diseases, such as lupus erythematosus (LE), systemic sclerosis (SS), and morphea, can significantly impact patients’ quality of life. Although disease activity can be controlled, residual scarring, atrophy, and hyperpigmentation can cause significant esthetic deformity, especially on exposed body parts such as the face and upper extremities. Therefore, it can negatively impact the patient’s psychological well-being, leading to low self-esteem, anxiety, and depression [16,17]. Moreover, various cosmetic procedures can mitigate the cosmetic sequelae of chronic inflammatory diseases, improving the appearance of skin lesions [16]. These include hyaluronic acid (HA)-based fillers, collagen stimulators, fat transfer, and laser therapies. Therefore, understanding the effects and safety of these cosmetic procedures in patients with autoimmune inflammatory diseases, as well as the risk of disease exacerbation, is crucial for guiding physicians [16,18,19].

This study revealed that patients who underwent cosmetic procedures were significantly more likely to report an adverse impact of their disease on their self-confidence. Low self-confidence and self-acceptance seem to be common features among patients with autoimmune diseases. Studies involving SLE patients showed that more than 50% of patients reported low self-acceptance, and only 16% reported high self-acceptance [16]. Cosmetic procedures led to a significant improvement in self-esteem among 46 participants [17].

In this study, the main reasons for not undergoing cosmetic procedures were fear of disease flare-ups, followed by fear of side effects. Such fears are supported by a few studies suggesting that collagen-based fillers may cross-react with anti-ds DNA antibodies in SLE patients, leading to the conclusion that fillers are contraindicated for patients with positive anti-ds DNA titers [18,20]. Conversely, another study assessed the occurrence of adverse events and disease flares associated with non-invasive and minimally invasive cosmetic dermatologic procedures, such as hyaluronic acid (HA) fillers, botulinum toxins, and laser procedures, among patients with systemic autoimmune disease. None of the 19 patients experienced disease exacerbation, and no changes in any titters or inflammatory markers were noted [18]. However, due to the small number of patients in this study, we cannot draw a concrete conclusion.

Multiple case reports showed no adverse reactions or disease exacerbation when using HA- and non-HA-based fillers in patients with lupus panniculitis/profundus; however, immediate post-injection nodularity is a frequent complaint, but it typically improves over time [21]. Overall, caution should be taken with injectables in patients with autoimmune diseases, and they should only be performed at times of stability and inactive disease. Additionally, a 2023 study evaluating the safety of esthetic procedures in patients with autoimmune inflammatory rheumatic diseases (AIRD) reported a low rate (15%) of adverse events [22]. The authors concluded that aesthetic procedures in patients with AIRD are associated with a risk of mild and transient site reactions, including those treated with biologic disease-modifying anti-rheumatic drugs (DMARDs) [22].

Regarding the performed procedures, hair removal by laser ranked highest, followed by fillers and Botox. A previous study from Iraq found that rhinoplasty was the most common intervention, followed by hair transplantation [23]. These differences in outcomes may be attributed to the fact that our patients have rheumatism and may tend to avoid more invasive surgical procedures such as rhinoplasty.

In this study, we found that the most frequently performed procedure was laser hair removal, which was undertaken by most subjects. Physicians often hesitate to use lasers and energy-based devices in patients with LE due to concerns about photosensitivity and photo-damage. However, studies show that pulsed-dye lasers (PDLs) at low fluences are usually well tolerated. Several reports and prospective studies have investigated the use of lasers for refractory malar erythema in active disease and for scarring in inactive disease. In a double-blind, randomized controlled trial of 48 discoid LE (DLE) lesions, PDLs significantly decreased erythema and improved physician global assessment scores [24,25]. Telangiectasias in SS are resistant to treatment and require multiple sessions [26,27]. Ablative lasers, such as carbon dioxide (CO_2_) lasers, have improved healing and reduced fibrosis in SS and morphea, potentially reducing joint contractures [24].

Abdominoplasty ranked third in this study as the most performed procedure. In a previous Saudi study from Al-Baha, abdominoplasty ranked second (16.7%) among the procedures performed at the hospital [28]. When looking at more invasive cosmetic procedures, one study compared abdominoplasty outcomes between normal healthy patients and patients with connective tissue diseases, including RA, SLE, Raynaud phenomenon, scleroderma, Sjögren syndrome, psoriatic arthritis, and mixed CTDs. The CTD group experienced higher rates of hematoma (4.5% vs. 3.0%; *p*  < 0.05), venous thromboembolism (VTE) (1.9% vs. 0.9%; *p*  <  0.05), and need for blood transfusion (13.0% vs. 7.7%; *p*  <  0.01) than the non-CTD group, respectively [29]. This raises the question and highlights the knowledge gap regarding whether patients with connective tissue diseases actually have an increased risk of adverse events when undergoing all types of cosmetic procedures, or whether the risk is limited to the more invasive procedures [11].

In this study, we found that undergoing cosmetic procedures was associated with female sex, college education, high income, and employment. Female sex increased the likelihood of undergoing cosmetic procedures by almost twelve times, while having a college education increased it by fourteen times [28]. In a previous study from Iraq, performing cosmetic procedures was considerably affected by sex and monthly income. The analysis revealed findings similar to ours regarding female sex, as the likelihood of undergoing cosmetic surgery was doubled among females; however, in contrast to our findings, the multivariate analysis showed no impact of education or monthly income on the procedures [18]. On the other hand, a Saudi study demonstrated that willingness to perform cosmetic procedures was not related to sex [30].

Clinically, this study sheds light on patients’ preferences for cosmetic procedures who have CTDs, underscoring the importance of proper education and counselling to prevent complications. Regarding study strengths, this study is the first to report the prevalence of cosmetic procedures in Saudi patients with rheumatological disorders. Limitations include a cross-sectional design, a patient-reported survey that may be subject to recall bias, a low response rate, and a limited number of participants.

## 5. Conclusions

This study highlights patient preferences and the need for appropriate education and counselling, while further research is required to better establish safety in this population. Reported cases in the literature found no adverse reactions when performing cosmetic procedures in patients with various autoimmune inflammatory diseases during periods of remission [19,21,22]. However, physicians should tailor their clinical judgement to individual cases, considering disease activity, immunosuppressive therapy, and procedure type [31]. Further research is required to determine the safety of cosmetic procedures in this patient population.

## Figures and Tables

**Table 1:** Demographics stratified by cosmetic procedure status.

		Did Not Consider/Undergo Cosmetic Procedure N = 120		Considered/Underwent Cosmetic Procedure N = 92		Total N = 212		*p*-Value
		N	%	N	%	N	%	
Age, mean (SD)		37	12	35	9	36	11	0.148
Sex	Female	106	88.3	91	98.9	106	88.3	0.003 *
	Male	14	11.7	1	1.1	14	11.7	
Nationality	Saudi	99	82.5	83	90.2	99	82.5	0.110
	Non-Saudi	21	17.5	9	9.8	21	17.5	
Residency	Central	72	60.0	51	55.4	72	60.0	0.816
	Western	13	10.8	13	14.1	13	10.8	
	Eastern	7	5.8	8	8.7	7	5.8	
	Southern	9	7.5	6	6.5	9	7.5	
	Northern	7	5.8	3	3.3	7	5.8	
	Other	12	10.0	11	12.0	12	10.0	
Marital	Married	57	47.5	47	51.1	57	47.5	0.605
	Unmarried	63	52.5	45	48.9	63	52.5	
Education	No education	0	0.0	0	0.0	0	0.0	0.043 *
	Elementary	7	5.8	1	1.1	7	5.8	
	Middle school	8	6.7	2	2.2	8	6.7	
	High school	30	25.0	18	19.6	30	25.0	
	College	70	58.3	61	66.3	70	58.3	
	Postgraduate degree	5	4.2	10	10.9	5	4.2	
Monthly income	(0–1000 SR)	30	25.0	13	14.1	30	25.0	0.036 *
	(1000–5000 SR)	34	28.3	16	17.4	34	28.3	
	(5000–10,000 SR)	27	22.5	28	30.4	27	22.5	
	(10,000–50,000 SR)	26	21.7	31	33.7	26	21.7	
	(>50,000 SR)	3	2.5	4	4.3	3	2.5	
Employment	Employed	38	31.7	42	45.7	38	31.7	0.037 *
	Unemployed	82	68.3	50	54.3	82	68.3	

* Significant according to a *p*-value of <0.050.

**Table 2:** Rheumatological illnesses stratified by cosmetic procedure status.

		Did Not Consider/Undergo Cosmetic Procedure N = 120		Considered/Underwent Cosmetic Procedure N = 92		Total N = 212		*p*-Value
		N	%	N	%	N	%	
Rheumatological disorder	SLE	53	44.2	47	51.1	100	47.2	0.122
	RA	47	39.2	24	26.1	71	33.5	
	Other inflammatory autoimmune disorders	20	16.7	21	22.8	41	19.3	
Patient-reported disease activity	Active	42	35.0	28	30.4	70	33.0	0.764
	Not Active	56	46.7	47	51.1	103	48.6	
	I don’t know	22	18.3	17	18.5	39	18.4	
Number of flares in the last three months	One	81	67.5	66	71.7	147	69.3	0.755
	Two	22	18.3	12	13.0	34	16.0	
	Three	8	6.7	7	7.6	15	7.1	
	More than 3	9	7.5	7	7.6	16	7.5	
Dermatological conditions related to the rheumatological disorder	No	62	51.7	49	53.3	111	52.4	0.818
	Yes	58	48.3	43	46.7	101	47.6	
Other comorbidities	No	78	65.0	62	67.4	140	66.0	0.716
	Yes	42	35.0	30	32.6	72	34.0	
Did your rheumatological disorder affect your self-confidence?	No	82	68.3	47	51.1	129	60.8	0.011 *
	Yes	38	31.7	45	48.9	83	39.2	
Taking medication for the rheumatological disorder	No	5	4.2	2	2.2	7	3.3	0.421
	Yes	115	95.8	90	97.8	205	96.7	
Antimalarials	No	62	53.9	37	42.0	99	48.8	0.094
	Yes	53	46.1	51	58.0	104	51.2	
Glucocorticoids	No	97	84.3	79	87.8	176	85.9	0.484
	Yes	18	15.7	11	12.2	29	14.1	
Biologics	No	78	67.8	70	77.8	148	72.2	0.115
	Yes	37	32.2	20	22.2	57	27.8	
Immunosuppressants	No	52	44.8	41	45.6	93	45.1	0.917
	Yes	64	55.2	49	54.4	113	54.9	

* Significant according to a *p*-value of <0.050.

**Table 3:** Factors that influenced consideration/undergoing of cosmetic procedures in patients with rheumatological disorders.

	Beta	Standard Error	Odds Ration	95% Confidence Intervals		*p*-Value
				Lower	Upper	
Female	2.486	1.045	12.019	1.550	93.175	0.017 *
Elementary school	0.560	1.330	1.750	0.129	23.703	0.674
Middle school	1.435	1.110	4.200	0.477	36.978	0.196
High school	1.808	1.083	6.100	0.730	50.984	0.095
College	2.639	1.201	14.000	1.329	147.429	0.028 *
(0–1000 SR)	0.082	0.450	1.086	0.450	2.621	0.854
(1000–5000 SR)	0.873	0.428	2.393	1.035	5.535	0.041 *
(5000–10,000 SR)	1.012	0.425	2.751	1.195	6.334	0.017 *
(10,000–50,000 SR)	1.124	0.833	3.077	0.601	15.740	0.177
Employed	0.595	0.287	1.813	1.033	3.181	0.038 *

* Significant according to a *p*-value of <0.050.

## Data Availability

All data generated or analyzed during this study are included in this published article. Further inquiries can be directed to the corresponding author.

## Supplementary Materials

The following supporting information can be downloaded at: https://www.mdpi.com/article/10.3390/healthcare14030378/s1, Supplementary Material S1: The English translated survey.

## Abbreviations

Rheumatoid arthritis (RA); connective tissue diseases (CTDs); systemic lupus erythematosus (SLE); Strengthening the Reporting of Observational studies in Epidemiology (STROBE); lupus erythematosus (LE); systemic sclerosis (SS); hyaluronic acid (HA); disease-modifying anti-rheumatic drugs (DMARDs); autoimmune inflammatory rheumatic diseases (AIRDs); pulsed-dye lasers (PDLs); randomized controlled trial (RCT); discoid lupus erythematosus (DLE).
