# Supplementary material for: The Prevalence and Effect of Cosmetic Procedures on Patients with Rheumatic Diseases: A Cross-Sectional Survey
Source: Healthcare (Basel). 2026 Feb 2;14(3):378. doi: 10.3390/healthcare14030378 (PMC12898049; doi:10.3390/healthcare14030378)
Supplement: Supplementary file 1 [file healthcare-14-00378-s001.zip › healthcare-4092673-supplementary.pdf]

Supplementary material S1. The English translated survey

1. Age (Years): (Quantitative)
2. Gender: (Male/Female)
3. Nationality: (Saudi/Non-Saudi)
4. City of Residence: (Al Baha/ Al Jouf/ Northern Border/ Riyadh/ Eastern Province/ Al Qassim/ Al Madina/ Makkah/ Tabouk/ Jazan/ Hail/Aseer/ Najran/ Outside KSA/Other)
5. Marital Status: (Single/Married/ Divorced/Separated/ Widowed)
6. Educational Level: (Non-educated/Elementary/Middle school/ Highschool/ College/ Post graduate degree)
7. Monthly Family Income: (0-1000/1000-5000/5000-10,000/10,000-50,000/ >50,000 Saudi Riyals)
8. Occupation status: (Full job/Part-time job/ Unemployed/ Student/ Retired)
9. Type of rheumatological disorder: (Systemic Lupus Erythematosus/Rheumatoid Arthritis/Ankylosing Spondylitis/Sjögren's Syndrome/Auto-Inflammatory Syndrome/Sarcoidosis/Kawasaki Disease/mucocutaneous lymph node syndrome/Takayasu Disease/Behcet's Disease (Behcet's syndrome)/Autoantibody Disease/Spondyloarthritis/Chondritis/Dermatitis/Inflammatory Myopathy/Tissue Inflammation/Uveitis/Polymyalgia Rheumatica/Multiple Sclerosis/Scleroderma/Gout/Other/Specify other)
10. How active is your rheumatological disease currently?: (Active / Not active / I don't know)
11. How many flare-ups do you experience in 3 months duration on average? (flare-ups requiring addition of medication, increase dose of medication or hospitalisation): (1 / 2 / 3 / More than 3)
12. The year: (Quantitative)
13. Have you had any skin involvement related to your rheumatological disease?: (Yes / No)
14. Were you prescribed any medication for your current illness (Rheumatic Disease)?: (Yes / No)

15. Medication type: (Abatacept (Orencia) / Antifibrotics (pirfenidone, nitedinib) / Antimalarials (hydroxychloroquine, chloroquine) (Plaqunel) / Apremilast / Azathioprine / 6-MP (Imuran) / Belimumab (Benlysta) / CD-20 inhibitors (rituximab, ofatu-mumab) / Cyclophosphamide / Cyclosporine / Denosumab (Prolya) / Glucocorticoids (prednisone, methylprednisolone) / IL-1 inhibitors (anakinra, canakinumab, rilonacept) / IL-6 inhibitors (tocilizumab, sarilumab) (Acetamra) / IL-12/23 inhibitors (ustekinemab, guselkumab) / IL-17 inhibitors (secukiumab, ixekizumab) / Intravenous immunoglobulin / JAK inhibitors (tofacitinib, baricitinib, upadacitinib) (Xeljanz) / Leflunomide (Arava) / Methotrexate / Mycophenolate mofetil / Mycophenolic acid (Cellcept) / Sulfasalazine / Tacrolimus (Prograf) / Thalidomide / Lenalidomide / TNF inhibitors (infliximab, adalimumab, golimumab, certolizumab, biosimilars) (Humera, Cemzia) / Steroid eye drops / Other / Specify other:)
16. Has your Rhumatological disease affected your confidence?: (Yes / No)
17. Were you ever diagnosed with any other diseases?: (Yes / No)
18. Type: (Interstitial lung disease / COPD/Asthma / Diabetes / Morbid obesity / Hypertension / Cardiovascular disease / Pulmonary hypertension / AKI or ESRD / Cancer / Organ transplant recipient / Immune deficiency / Inflammatory bowel disease / Liver disease / Neurological or neuromuscular disease / Down’s syndrome / Other / Specify other)
19. Were you perscribed any medication for your disease? (Yes/No)
20. Were you ever diagnosed with a psychiatric disease? (Yes/No)
21. Type: Depression / Psychosis / Anxiety / Neurodevelopmental disorder / Obses-sive Compulsive Disorder / Bipolar Disorder / Post-traumatic Stress Disorder / Eating Disorder / Personality Disorder / Sleeping Disorder / Other / Specify other
22. Were you perscribed any medication for your psychiatric disease? (Yes=/No)
23. Have you ever considered undergoing cosmetic dermatological procedure? (Not relating to rheumatological diseases treatment): (Yes=/No)

24. If yes, which of the following cosmetic dermatological procedures did you consider undergoing? (Choose all that apply. If the procedure is not listed, kindly choose "Other" and mention it in the box below): (Considered Fillers / Considered Botox / Considered laser hair removal / Considered other dermatological procedures / Specify other)
25. Have you ever considered undergoing a surgical cosmetic procedure? (Not relating to rheumatological diseases treatment) (Yes/No)
26. If yes, which of the following surgical cosmetic procedures did you consider undergoing? (Choose all that apply. If the procedure is not listed, kindly choose "Other" and mention it in the box below) (Considered Rhinoplasty / Considered face lift / Considered blepharoplasty / Considered breast augmentation / Considered breast reduction / Considered breast lift / Considered liposuction / Considered Abdominoplasty / Considered Brazilian butt lift / Considered other surgical cosmetics procedures / Specify other)
27. Reason: (lack of confidence in appearance / Convinced by social media / Suggestion from friends and fam-ily / Sugestion from Doctor / Other reason for considering cosmetic procedure / Specify other)
28. If you have considered undergoing a cosmetic procedure but have not done so what were the reasons? (Choose all that apply. If not listed, kindly choose "Other" and mention it in the box below. (Afraid of flare up of rheumatological disease / Afraid of side effects of cosmetic procedure / Was told by rheumatologist to avoid cosmetic procedure / Physician (dermatologist/surgeon) refused to perform cosmetic procedure / Other reason for considering, but NOT undergoing a cosmetic procedure / Specify Other)
29. Have you ever undergone any cosmetic procedures (Dermatological or surgical) before your diagnosis with your rheumatological disease?: (Yes=/No)
30. If yes, which of the following procedures did you undergo before your diagnosis with your rheumatological disease? (Choose all that apply. If the procedure is not listed, kindly choose "Other" and mention it in the box below): (Underwent "Botox" before diagnosis / Underwent "Fillers" before diagnosis / Underwent "Laser hair removal" before diagnosis / Underwent "blepharoplasty" before diagnosis / Underwent "Rhino-plasty" before diagnosis / Underwent "Breast augmentation" before diagnosis / Underwent "Liposuction" before diagnosis / Underwent "Abdominoplasty" before diagnosis / Underwent "Brazil-ian Butt Lift" before diagnosis / Underwent "Other Procedure" before diagnosis / Specify "Other")
31. If yes, how many separate times did you undergo each procedure before your diagnosis with rheumatological disease? (Quantitative)
32. If yes, did you experience any complications after any one of these procedures? (1 month after the procedure): (Yes/No)
33. If yes, did any of these complcations cause hospital admission or antibiotics? (Yes/No)
34. Have you undergone any cosmetic procedure after your diagnosis with rheumatological disease? (Yes/No)
35. if yes, how did you feel after it? Choose all that apply. If not listed, kindly choose "Other" and mention it in the box below): (Confident / Insecure / Regret / Neutral / other / if yes, how has the cosmetic procedure affected your confidence? / positively / negatively / no effect)
36. If yes, was your rheumatological disease controlled before undergoing the procedure? (Yes/No)
37. If yes, what were the medications used for disease control before undergoing the procedure? (Choose all that apply. If the medication is not listed, kindly choose "Other" and mention it in the box below): (did not use any drugs / abatacept (orencia) / Antifibrotics: including pirfenidone, nitedinib / Antimalarials: including hydroxychloroquine, chloroquine (Plaqunel) / Apremilast / Azathioprine / 6-MP (Imuran) / Belimumab / CD-20 inhibitors: including rituximab, ofatumumab / Cyclophosphamide / cyclosporine / Denosumab (Prolya) / Glucocorticoids: including prednisone, methylprednisolone / other / specify others)
38. if yes, did you stop any of your medications for your rheumatological disease before undergoing the procedure? (Yes/No)

39. Which of the following cosmetic procedures did you undergo after your diagnosis with a rheumatological disorder? (choose all the apply. If not in the list please choose "other" and specify): (Underwent fillers after diagnosis with rheumatological disorder? / Underwent botox after diagnosis with rheumatolog-ical disorder? / Underwent laser hair removal after diagnosis with rheumatological disorder? / Underwent fractional laser after diagnosis with rheumatological disorder? / Underwent laser mole removal after diagnosis with rheumatological disorder? / Underwent Chemical peel after diagnosis with rheumatological disorder? / Underwent Hyaluronic Acid/ Profhilo Injections after diagnosis with rheumatological disorder? / Underwent Liposuction after diagnosis with rheumatological disorder? / Underwent Rhinoplasty after diagnosis with rheumatolog-ical disorder? / Underwent Blepharoplasty after diagnosis with rheumatological disorder? / Underwent Brachioplasty after diagnosis with rheumatological disorder? / Underwent Abdominoplasty after diagnosis with rheumatological disorder? / Other / Specify other)
40. If yes, how many separate times did you undergo each procedure after your diagnosis with rheumatological disease? (Quantitative)
41. How many flare-ups did you experience in the 3 months prior to the cosmetic procedure? (flare-ups requiring the addition of medication, an increased dose of medication or hospitalisation): (1/ 2/ 3/ More than 3/ No flare-ups)
42. Did you experience any increase in rheumatological disease activity within a month after undergoing cosmetic procedure? (Yes/ No)
43. If yes, how many of these increases in disease activity require a new drug, an increase in the dose of the current drug or hospital admission, within a month after undergoing cosmetic procedure? (1/ 2/ 3 / More than 3/ didn't require/
44. Did you suffer from any other side effects (not related to your rheumatological disease) after undergoing the cosmetic procedure (within a month after undergoing the procedure): (Yes/ No)
45. If yes, did any of these side effects require hospital admission of the use of antibiotics? (Yes/ No)
